# What is in a Name? Parent, Professional and Policy-Maker Conceptions of Consent-Related Language in the Context of Newborn Screening

**DOI:** 10.1093/phe/phz003

**Published:** 2019-05-04

**Authors:** Stuart G Nicholls, Holly Etchegary, Laure Tessier, Charlene Simmonds, Beth K Potter, Jamie C Brehaut, Daryl Pullman, Robin Z Hayeems, Sari Zelenietz, Monica Lamoureux, Jennifer Milburn, Lesley Turner, Pranesh Chakraborty, Brenda J Wilson

**Affiliations:** 1 School of Epidemiology and Public Health, University of Ottawa and Ottawa Hospital Research Institute (OHRI); 2 Clinical Epidemiology Unit, Faculty of Medicine, Memorial University, St John’s, Newfoundland and Labrador; 3 Newborn Screening Ontario, Children’s Hospital of Eastern Ontario; 4 Health Research Unit, Faculty of Medicine, Memorial University, St John’s, Newfoundland and Labrador; 5 School of Epidemiology and Public Health, University of Ottawa; 7 Community Health and Humanities, Faculty of Medicine, Memorial University, St John’s, Newfoundland and Labrador; 8 Program in Child Health Evaluative Sciences, The Hospital for Sick Children and The Institute of Health Policy, Management and Evaluation, University of Toronto; 12 Provincial Medical Genetics Program, Eastern Health, St John’s, Newfoundland and Labrador; 13 Newborn Screening Ontario, Children’s Hospital of Eastern Ontario and Department of Pediatrics, Faculty of Medicine, University of Ottawa; 14 Division of Community Health and Humanities, Faculty of Medicine, Memorial University of Newfoundland

## Abstract

Newborn bloodspot screening programs are some of the longest running population screening programs internationally. Debate continues regarding the need for parents to give consent to having their child screened. Little attention has been paid to how meanings of consent-related terminology vary among stakeholders and the implications of this for practice. We undertook semi-structured interviews with parents (*n* = 32), healthcare professionals (*n* = 19) and policy decision makers (*n* = 17) in two Canadian provinces. Conceptions of consent-related terms revolved around seven factors within two broad domains, *decision-making* and *information attainment*. *Decision-making* comprised: parent decision authority; voluntariness; parent engagement with decision-making; and the process of enacting choice. *Information ascertainment* comprised: professional responsibilities (including disclosure of information and time to review); parent responsibilities; and the need for discussion and understanding prior to a decision. Our findings indicate that consent-related terms are variously understood, with substantive implications for practice. We suggest that consent procedures should be explained descriptively, regardless of approach, so there are clear indications of what is expected of parents and healthcare professionals. Support systems are required both to meet the educational needs of parents and families and to support healthcare professionals in delivering information in a manner in keeping with parent needs.

**Research Highlights**

- Current lack of attention given to meaning of consent-related terminology.
- The meaning of consent-related terminology varies among stakeholders.
- Meanings of consent-related terms come with practical implications.
- Identifies seven key factors that distinguish meaning of consent-related terminology.

## Introduction

Newborn bloodspot screening (NBS) is a global activity, with some of the largest and longest running population screening programs in the world (Green *et al.*, 2006). The growing number of conditions included within NBS programs has spawned debate regarding the primary purpose and supplemental benefits of screening (Ross 2006; Nicholls, Wilson *et al.*, 2014), as well as the need (or not) for explicit parental consent (Newson 2006, 2011; Fost 2016; Kelly *et al.*, 2016). Newborn screening has received renewed interest due to several research studies exploring the interest in, and utility of, whole genome sequencing as part of NBS (Botkin and Rothwell 2016; Johnston and Jeungst 2018). Different screening programs have different approaches to consent, including opt-in and opt-out models (AAP Newborn Screening Task Force 2000; Hanley 2005; Therrell *et al.*, 2006; UK Newborn Screening Programme Centre 2008; Ross 2010; Buchbinder and Timmermans 2011, 2012).

However, there is a readily acknowledged inconsistent use of terminology to describe these models (Hargreaves *et al.*, 2005a). For example, the Canadian model has been variously described as ‘implied consent’ (Miller *et al.*, 2010) and ‘standard of care’(Araia *et al.*, 2012), while opt-in approaches are often described as informed ‘choice’, ‘consent’ or ‘dissent’ (Hargreaves *et al.*, 2005a).

Not only does the *use of terms* differ but *definitions of these terms* also differ across the screening literature. Within the empirical literature on prenatal screening, for example, the conception of what constitutes an informed choice is debated. Dormandy *et al.*, in their influential work developing the Multidimensional Measure of Informed Choice, define an informed choice as ‘one that is based on relevant knowledge, consistent with the decision-maker’s values and behaviourally implemented’, emphasizing the role of value consistency as a core component (Dormandy *et al.*, 2002). Others, such as Summers, suggest equivalency between terms, stating that ‘Informed choice or decision making generally involves three components: information, comprehension, and voluntary choice’ (Summers, 1994). Biesecker and colleagues have further argued that informed choice can be measured as an ‘integrated decision’ requiring knowledge and values clarity (Biesecker *et al.*, 2013), again suggesting that a choice and decision are equivalent. However, others distinguish an informed choice from an informed decision. van den Berg *et al.*, for example, argue that ‘A choice refers to the end product of a decision, whereas a decision refers to the process of choosing between alternatives, preceding that choice’, and thus the additional deliberative aspect differentiates an informed choice from an informed decision (van den Berg *et al.*, 2006). Yet here there also appears to be no consensus, with van den Berg and colleagues themselves identifying nine different definitions of informed decision in the literature. Each of these definitions place varied emphasis on the informed nature of the choice, voluntariness, comprehension, the enactment of decision, the totality of possible courses of action evaluated as well as the types of information that need to be evaluated (van den Berg *et al*., 2006). Despite these noted variants, the definitions tend to reflect only the aspects related to the decision by the individual and are relatively silent on the responsibilities of those involved in the imparting of information to the individual.

Others have similarly differentiated informed choice from informed consent. Jepson and colleagues, for example, have differentiated informed consent and informed choice on the basis that consent reflects a more active process of decision-making (with contact and discussion with a health professional) than an informed choice (Jepson *et al.*, 2005). In contrast to the above literature, this definition explicitly includes roles and responsibilities for the healthcare professional and implicates discussion as a key component of the informed consent process.

Other aspects of debate have included the content required to be disclosed by healthcare professionals and the extent to which disclosed information needs to be understood in order to constitute an informed consent (see, for example, Faden and Beauchamp 1986; Beauchamp and Faden 1995; Manson and O’Neill 2007; Wilson 2007; Dawson 2009; Walker 2012; Manson 2013). Sreenivasan (2003) and subsequently Bromwich (2015) argue that information disclosure must be disentangled from subsequent understanding of the disclosed information: it may be a requirement that certain information has to be disclosed, but this *in and of itself* does not create a requirement that the individual understand this information. Further, Walker (2012) explicitly differentiates informed choice and informed consent on the basis of the type and/or content of information that needs to be disclosed by the healthcare professional.

With regard to NBS, the topic of information disclosure under differing consent approaches has only recently been discussed. In their work, Potter *et al.*, incorporate parent engagement in the decision-making process as a key factor associated with the information to be disclosed under different consent models. The authors suggest that there may be less expectation for parents to engage in decision-making under certain consent models (such as implied consent) than under an informed consent model. Further, where there is no need for parents to make a decision and there is less expectation of engagement, this may be associated with differences in the information provided to parents (Potter *et al.*, 2015).

Despite the ongoing philosophical debate, and nascent consideration with the NBS literature, the extent to which stakeholders in newborn screening see varied consent-related terminology as having substantive implications for practice has not been explored. We contend that promoting effective consent processes requires an examination of how consent-related terms are understood from multiple perspectives including: (i) policy makers who presumably stipulate consent standards that are meant to be reflected in practice; (ii) health professionals implementing consent procedures; and (iii) individuals making a decision on behalf of the child.

An empirical social science perspective is well suited to identifying the role that social structures, social routines and social patterns or values play in the organization and delivery of a healthcare system in general, and for a complex process like NBS in particular. In order to understand the consent process for NBS we need to understand not only what an ideal consent process might look like but also how this process might be operationalized in the real world, including identifying potential barriers or facilitators to that ideal (Juth and Munthe, 2012). For example, how does an institutional structure that embodies the expectation that babies are born in a hospital affect the nature and extent of information on NBS provided to parents? Do parents perceive NBS as mandatory, as an expectation or as a voluntary service? How might this impact the nature of ‘consent’? Institutional settings may generate ‘coercive situations’ (Faden and Beauchamp, 1986) where a choice is available (and hence voluntary), but where subtle pressures may drive a choice in a particular direction (Nicholls, 2012). Would the consent process play out differently in the context of midwife-led care, or for a home birth outside the hospital setting? Moreover, how does the setting affect the mechanics regarding what information is disclosed, by whom and when, and how do these aspects affect the nature of any consent given?

Understanding how stakeholders conceptualize consent-related terminology, and the practical implications of these conceptions, may illuminate deficiencies in current practices and identify reasons for this. It may also identify potential improvements for practice.

This study was designed to explore meanings and perceptions of consent-related terminology in the context of NBS. Specifically, our research questions were (i) how are different consent-related terms conceptualized and interpreted by key stakeholders in NBS? and (ii) how do stakeholders differentiate consent-related terms and consent-approaches? Based on our interviews we characterize the distinguishing features of consent terms and approaches as identified by parents, healthcare professionals and policy decision makers. In so doing, we highlight areas where greater policy clarity or on-the-ground interventions might be beneficial.

## Materials and Methods

We undertook semi-structured interviews with key stakeholders involved in the NBS program, namely parents, healthcare professionals and policy decision makers. The full study protocol is published in detail elsewhere (Nicholls, Tessier *et al.*, 2014).

### Identification and Recruitment of Participants

We carried out the study in parallel at two research sites: Ontario (ON) and Newfoundland and Labrador (NL), Canada. These provinces differ in their screening program composition and organization; ON has the largest screening program in Canada, screening over 140,000 samples per year for 29 conditions; conversely, NL screens for only six conditions, with roughly 4500 births per year. Both programs ostensibly operate under similar consent procedures which are generally reflective of those reported across Canada (Miller *et al.*, 2010; Araia *et al.*, 2012; Etchegary *et al.*, 2016).

Parents were eligible for inclusion if they were over 18 years of age, their child had been offered NBS within either ON or NL during the past year, they currently resided in ON or NL, and could converse fluently in English or French. All parents were identified through records held by each provincial screening program on the basis of screening result (normal, false positive, true positive or declined). Following review by a genetic counselor or geneticist to ensure eligibility, ON parents were contacted in writing by a member of the clinical team. As ON records only maternal details all contacts were addressed to the mother. Due to very small numbers at the NL site, parents offered NBS received a phone call from the geneticist who provided care during the screening process to gauge interest in the study. Those indicating interest were mailed study information. Parents who declined NBS at the ON site were contacted through their healthcare professional and were provided study materials if interested. All participants were offered a small financial compensation for their time.

Healthcare professionals were purposively sampled and were eligible for inclusion if they were involved as submitters of bloodspot samples to the provincial screening program or were actively involved in the provision of education regarding NBS. Purposive sampling is a deliberate non-random approach in which individuals or groups are sought on the theoretical basis that holding a particular characteristic may be associated with a particular perspective (Bowling 2004). Just as we sought parents with different experiences on the assumption that the nature of their experiences might affect their attitudes or perspectives on consent, so we sought different healthcare professionals involved in the consent process. In particular, we sought out both hospital-based staff and community-based midwives. Previous work in ON indicated that one-third of cases in which infants are not screened have midwife involvement, although only 10 per cent of births in the province are under midwife care, suggesting a higher rate of parents potentially declining screening (Milburn *et al.*, 2012). This, we felt, may reflect a differing opinion or experience regarding the consent processes or the notion of directiveness, with midwives potentially being less directive regarding screening (Miller *et al.*, 2010). Participants were identified through screening reports, as well as existing professional and organizational networks. All healthcare professionals were contacted in writing by a member of the clinical team.

In both provinces, policy decision makers were eligible for inclusion if they were currently serving, or had served, as a member of an NBS advisory body. A snowball sampling approach was also used to supplement the initial sampling from committee membership lists (Bowling 2004; Adair *et al.*, 2009). All invitees received a mailed invitation to take part in the study.

In all cases, individuals identified as eligible received an invitation along with a response slip, a stamped return envelope and a copy of the information sheet and consent form. Participants indicated willingness by returning the reply slip to the study team. An interview date was then arranged. In all cases, consent was confirmed verbally on the date of the interview. Recruitment continued until theoretical saturation had been achieved—that is, no new themes were emerging from the data.

### Data Collection

Semi-structured interviews were conducted either in person or by telephone. Initial discussion drew on participants’ own experiences of the consent process for NBS. Following this, participants were provided information about existing variation in practice and debate as to the need or not for informed consent. This included a brief overview of how screening proceeds in different jurisdictions. Specifically, participants received basic information about different screening programs using archetypal examples of mandatory, opt-in and opt-out approaches. Participants were then asked to describe what ‘informed consent’ meant to them and were further probed with respect to what needed to happen for an informed consent to occur. Participants were asked to explicitly contrast an informed consent approach with provided alternatives (mandatory screening, implied consent) as well as any approaches that had been spontaneously generated by the participant. Each time they were asked to identify differences (if any) between the approaches as well as the significance of perceived differences. Participants were then asked to select a preferred approach and say why that was preferred over other options. Copies of the interview schedule are provided as Supplementary Materials.

Interviews were audio-recorded and transcribed verbatim. During the transcription process potentially identifying comments and remarks were de-identified. Final transcripts were imported into qualitative data analysis software to assist with analysis.

### Data Analysis

The goal of this analysis was to compare responses within and across respondents and not to develop a detailed understanding of individual experiences of consent (see Etchegary *et al.* (2016) for an overview of parent and healthcare professional experiences). The evaluation of transcripts followed a thematic analysis approach (Boyatzis 1998; Braun and Clarke 2006). Thematic analysis shares many features with other methods of qualitative analysis in so far as textual data is analyzed in an inductive manner, with the generated themes grounded in the data as opposed to using an *a priori* coding scheme (Braun and Clarke 2006). Transcripts were coded and labeled by a member of the team (S.G.N.) in an inductive manner with textual elements coded iteratively; individual codes were developed using the constant comparison method alongside the interviews, then reviewed or revised in light of emerging data (Strauss 1987). This initial thematic analysis was assessed by a second analyst (H.E.) who reviewed all transcripts for coding consistency and accuracy of themes, with subsequent discussion between analysts. Final analyses were then presented to the broader team for comments and further discussion. Themes were then further revised until a final set was derived.

The study was approved by the Ottawa Health Science Network Research Ethics Board, the Children’s Hospital of Eastern Ontario Research Ethics Board and the NL Health Research Ethics Authority. Interviews were conducted between June 2014 and May 2015.

## Results

Numbers of participants by role and province are presented in Table 1. The presence of standing committees in ON likely contributed to the preponderance of policy decision makers from that province. A lack of parental declines in NL precluded interviews there.

**Table 1.** Interview participants

Stakeholder group	Ontario (*N*)	Newfoundland and Labrador (*N*)	Total (*N*)
Parents: Screen negative	5	8	13
Parents: True positive	6	3	9
Parents: False positive	5	2	7
Parents: Decline	3	0	3
Health Professional	15	4	19
Policy decision-maker	15	2	17
Total	49	19	68

Overview of interview participant demographics.

### Lexical Variation

Throughout the interviews participants used a variety of terms, both spontaneously and after prompting, to describe different consent approaches. These included ‘informed consent’, ‘informed choice’, ‘implied consent’, ‘presumed consent’, ‘assumed consent’, ‘valid consent’, ‘reverse consent’ (for opting out) as well as ‘standard of care’. Within every stakeholder group, participants used common terms to mean different things. For example, when discussing informed consent, some participants described an extensive process while others discussed a more limited range of requirements. Similarly, approaches that were referred to under different terms were similar in their practical description. Hence, while some described more cursory discussion of information as part of an informed consent model, others described similar interactions as indicative of an implied consent or assumed consent approach.

Stakeholder groups varied in their outlook. For example, policy decision makers often invoked larger societal discussions of consent, or broader policy contexts in which consent fit, while parents tended to discuss consent more within the specific interaction with their healthcare professionals. While the use and conceptions of consent-related terminology varied, we did not identify systematic differences in the use of terms between stakeholder groups.

Despite the noted variation in language, we identified seven important factors which participants used to distinguish between *different* consent approaches. These dimensions fell within two broad domains: (i) *decision-making*, comprising: parental decision authority; voluntariness; parental engagement with decision-making; and the process of enacting choice and (ii) *information ascertainment*, comprising: professional responsibilities which entailed disclosure of information and time to review; parental responsibilities; and the need for discussion and understanding prior to a decision.

### Decision-Making: Parental Decision Authority

A recurring aspect of discussion with participants was whether a particular approach gave parents the authority to make decisions for their child, and to what degree. Comparisons—particularly between mandated and alternative approaches—drew particular contrasts along lines of decision-making authority, and specifically about which actors were legitimate decision makers.

“Well I guess like I said before mandated just means you don’t have a choice the child is tested I guess they don’t even, I guess they tell you it’s going to happen but you don’t have a choice in the matter. Whereas implied consent you know as long as you don’t say no they can go ahead with it. I guess informed consent actually gives you a choice whether or not you want the testing completed or not.” *P34, Parent, false positive, NL*

While mandated screening and informed consent approaches were most notably contrasted on this dimension, other terms—such as implied consent—were also felt to convey varied notions of parental authority to make a decision on behalf of their child:

“I guess implied consent would be kind of given to this grey area where, you know, people could interpret it that it’s not optional – that it is something that you have to do.” *P46, Parent, Screen Negative, ON*

### Decision-Making: Voluntariness

While parental decision authority implies that screening is something parents may accept or decline, a related aspect upon which participants differentiated approaches was the degree to which parents were pressured or experienced resistance to decisions. For some, the language of ‘consent’ indicated an expectation of agreement, whereas ‘choice’ was less directive:

“Um … but informed consent would be – I’m telling you all these things with the understanding that you’re going to consent at the end of that, whereas informed choice would be – I’m telling you all of these things and really, it’s still up to you. You still have a choice as opposed to, you know – this is a hospital protocol. You have to do it, but I want you to be informed about doing it. Versus - No, this is actually voluntary, but I want you to have all of the information so you can make an accurate decision. So I would say the two of them are still a little bit different to me.” *P4, Healthcare Professional, ON*

Further, while some approaches did—in theory—allow parents to decide (and hence were voluntary), this was not necessarily done in the same manner across approaches. For example, when one respondent was asked to expand as to which of the consent models discussed represented how they understood the NBS process, they indicated that it would be ‘standard of care’, but this was not non-directive:

“So, I … I think, uh, it is standard of care […] a woman still has … does have the right to decline, and if it was mandatory, she would not. She does have the right to decline but she has to jump through some hoops […] I think it’s a little bit coercive, for if a woman declines, […] there’s two documents: one document goes to [screening program] signed by the woman to say that she declined, and another one that goes—a separate document required by the hospital legal services, that stays in the hospital, that says she declined. So, that’s … that’s, to me, that is a bit … a bit coercive and, but certainly some people do sign them.” *P52, Policy Decision-Maker, ON*

Thus, while various approaches to consent may allow parents to have the ultimate authority to accept or decline screening, these were not simply binary; models that provided parents the authority to decide had a degree of gradation with respect to the amount of pressure applied to parents—specifically if they indicated an intention to decline.

### Decision-Making: Parental Engagement with Decision-Making

Another decision-making factor was the perceived expectation for parents to engage with the decision-making process. For some, informed consent indicated an expectation of engagement in the decision-making process. When asked what the term informed consent meant, one participant responded:

“Well it would mean that the mom and dad or whoever are the caregivers of the baby would know all the risks and benefits of this test. So someone would explain to them you know what is being tested. What is the blood being tested for? What are the potential outcomes of a positive test? Who is going to follow up that positive test? Are there any potential risks of having that test done and you know then they actually make a decision but they have, someone is giving them the information about what the test is for. So it’s informed, they’re knowledgeable about what the test is for and they’ve made a decision about whether they want it done or not.” *P8, Healthcare Professional, NL*

This expected engagement appeared to be associated with the expected authority—if the default is to assume agreement unless otherwise stated, then the expectation for engagement was lessened.

“And, um, implied consent is that they don’t really ask; they just assume that it’s accepted and they go along with whatever, without really giving any information to make a choice, or to let you know that it even has a choice […] And the same thing with presumed consent. I would assume implied and presumed are basically the same?” *P49, Parent, Declined, ON*

Despite this, the expectation that informed consent requires a deliberated decision was not universal. Other participants saw informed consent as not necessarily requiring engaged decision-making, but would be sufficient with a less involved process:

“Informed consent well to me it sounds like the person is provided information on the procedure or the testing and basically they just agree to it.” *P35, Parent, False Positive, NL*

### Decision-Making: Process of Enacting Choice

A further aspect upon which participants defined consent models was the way in which parents enacted their choice or decision. This reflected both whether the process required an active role as well as the form required.

“Implied, to me, is when an out-patient walks in. That’s implied consent. They’ve handed me their requisition, they’re letting me know ‘I want you to do this.’ So that’s the implied consent. Presumed consent is what I think of when we go to the patients’ floors –we’re presuming there’s consent there because the physician or the nursing has told them that we’re coming. And that could be for any procedure that we have to perform on them.” *P16, Healthcare Professional, ON*

Yet while the process—either through a physical act of showing an arm or coming to hospital—was seen as important, for some participants the mode of enacting a choice was not substantially important to differentiating approaches; both verbal and written consent to screening were acceptable forms for informed consent. Other respondents indicated that approaches did in fact differ with respect to how the act of indicating consent occurred. Written authorization, for example, was a commonly cited example associated with informed consent, but not required with other models. For some, the requirement for a written consent was explicitly linked to their belief that understanding (of the decision at hand) was a necessary component of an informed consent. As one parent put it:

“Implied consent seems nice but I don’t know. I think that you should have to sign a form then maybe that would give the people you know it would give the parents a little bit more information about what they’re actually, what’s actually being done. Then even if they just sign it and they may do some of their own research if they want or they may actually ask more questions you know. Because if it’s just kind of a verbal, is this okay, there’s so much stuff going on in the hospital you’re like, yeah, yeah, yeah of course you want what’s best for your baby right.” *P36, Parent, Screen Negative, NL*

This was related back to the responsibilities of healthcare professionals:

“Hmm … I mean, I guess because I don’t use implied or, um … in consent, I don’t think about it in that way very often. […] the consents I use for [screening], are […] we hope they are informed in that the care provider—the screener—has, has, you know, the information that they’re supposed to relay to the parent. Now in both cases, the parent doesn’t actually sign anything. And maybe that’s that implied … that’s sort of another piece in there … it’s the care provider has checked off on the box “consent has been received.” That’s verbal consent, […]. So, in both those cases, we are getting a verbal consent from the family, um, that they understand what, um, uh, what is being done. They understand the risks (I mean in this case there aren’t really any). They understand what will be done with the info—what will be done with the findings. And that, […] and then the care provider just checks off on the box, “yes.”[…] Um, now … again, that relies … that’s a consent that relies heavily on, um, the integrity of the care provider to ensure that they genuinely got a verbal consent […].” *P51, Policy Decision-Maker, ON*

“[…] I think we’re moving progressively to documentation of choice. So we did perceive this as a risk and educational issue. So one - were people actually talking to people about the choice. Right? So having included a decline form as part of our actual screening card. Right? I think reminds people that there’s a choice. Right? You know, people can decline. Um, and it’s also documentation …” *P56, Policy decision maker, ON*

In some instances legal responsibility was noted for requirements to gain written authorization for informed consent. As one, slightly exasperated, respondent indicated, they would favor written authorization over other forms of authorization, largely from a defensive medicine position:

“[Sigh] because we have a hundred lawyers working for us […] I know, I think we tend to err on the side of caution, absolutely. And, um, and, and generally our consents aren’t … as I’ve said earlier … generally most of our consents are not just about, um, “can I do this to you?” but they are “what am I going to do with the information?”” *P51, Policy Decision-Maker, ON*

Consequently, the process by which any consent was enacted related to the decision authority of the parents, but also professional responsibilities to obtain consent and perceived legal responsibilities.

### Information Ascertainment: Professional Roles and Responsibilities

The differentiation of approaches based on parental engagement fed into further discussions regarding how different approaches generated differing responsibilities for parents and healthcare professionals. The notion of responsibility was reflected in two main facets: first, responsibility in a devolved sense where parents had devolved responsibility of decision-making to professionals or professional bodies through governance decisions. For example, one healthcare professional, when discussing implied consent, took the implied aspect to be implicit consent to professional decisions of best interests for the child. However, the dominant discourse regarding professional responsibilities pertained to questions of what was required to be done, as opposed to who had authority.

#### Professional responsibilities: disclosure of information

A strong narrative was the responsibilities of professionals with respect to information disclosure. As indicated in the earlier quotation on parental engagement, perspectives varied regarding the level of disclosure needed. Notably, when participants were asked explicitly to consider the language of ‘implied consent’ a recurring thought was that this potentially reduced professional responsibilities to provide information to parents:

“I find it terrible. To me, having something, as “implied consent” sort of sounds like ‘we don’t have to do our due diligence and tell you about it, we just have to assume that you’re gonna say yes unless you tell us no’. […] Because then you’re putting the onus for the information on the mothers versus the healthcare professionals telling you what’s going on.” *P1, Parent, Screen Negative, ON*

For this participant, the onus should not be something foisted on parents, yet an implied consent was felt to place that information-seeking responsibility on the parents rather than the professional’s responsibility to inform. Others felt the models differed with respect to information provision and explicitly contrasted informed consent and implied consent with respect to the information provision for parents:

“In informed I guess the benefit of informed is that [it] ensures that people are at least given some information about the test whereas with implied there isn’t the same process to get that information to the patient.” *P43, Parent, Screen Negative, NL*

“I guess informed consent requires, uh, an in-depth conversation. I guess, totally in a way, the clinician has to make a judgment as to whether you’re fully aware of all the facts—and all the risks and all the benefits, and if you can fully comprehend those, and I think implied probably doesn’t have that … that much of a requirement, I would say to that.” *P46, Parent, Screen Negative, ON*

“I think what I would take by implied, is that there was some explanation given to parents about the test being made to their baby, and that unless they made a very clear refusal, for it to be done, that consent was implied and, and implied in the sense that there’d been an explanation given. I think … I don’t know whether, in fact, there were … would have been settings where no explanation was given, and therefore it was … you could call it presumed … that, it would be presumed that parents understood—or assumed, probably wrongly, that parents understood exactly what was going on.” *P53, Policy Decision-Maker, ON*

However, a contrasting opinion was that the form of consent approach did not modify the responsibility on healthcare professionals to provide information. Indeed, even when discussing mandated screening both parents and healthcare professionals discussed the need for information provision for parents. As one parent indicated:

“So making the information available, to me, is more important than saying everybody has to sign off on this because by you saying everybody has to sign off on this … you know, look, the reason why they’re saying sign off on it is because you can … now you can guarantee that obstetrician has talked to that patient, or that doctor has talked to that patient, obviously because they’ve had to sit down and explain everything, and that’s what the signature says.” *P26, Parent, Screen Negative, ON*

Perspectives regarding parent engagement in decision-making and professional responsibilities for disclosure also informed views regarding the perceived content of information that was required to be disclosed for any consent to be given. When participants discussed the disclosure of information across consent approaches, they openly reflected on the content, level of detail and the format of the information. This was particularly notable in descriptions of informed consent where some participants viewed an informed consent to require extensive information:

“I think the diseases that are being tested for that should be there and probably written out so that they know what’s being tested for because parents especially when they’ve been in labour for a day or two and then maybe had a section like they’re not thinking properly. It might even be important to tell them that this is going to be done you know even before the baby is born. So the informed consent is not you know at the moment that you’re about to take the blood but prior to their admission to hospital. That when your baby comes in the hospital these are the procedures that we would, that are recommended to have happen to your baby just as we recommend vitamin K and eye ointment. […] So I guess 1) yes it’s probably important to be written; 2) it should be prior to the delivery of the baby and not just at the moment of the blood being taken; 3) it should be for which diseases are being sought you know looked for and maybe 4) you know what are the risks of a false positive and what happens if a positive result happens, what’s the procedure.” *P8, Healthcare Professional, NL*

In contrast, others viewed an informed consent to require information to be provided only in general terms or were less stringent on how extensive the discussion needed to be. The lack of consensus around appropriate content was reflected by one participant who indicated that consent—as in agreement—may not necessarily be informed. When pressed on what would constitute ‘informed’ in the context of a consent process, they noted:

“[…] Right. Well, you know, and informed is … its relative, right? So in … how much information? Whose information? What evidence are you using? Like, I mean it’s all … it’s all malleable.” *P5, Healthcare Professional, ON*

#### Time

Participants differentiated consent approaches through time-related issues. This included the timing of discussion and information disclosure, the timing of consent or decision itself, but also the availability of time in which to make a decision or choice. For example, some participants differentiated consent approaches on the basis of parents being afforded time to review information.

“Well, I mean, I wouldn’t mind being given information, so long as I have time to ask the questions if I need clarification, um, or trying to read it, you know. If they just hand me the information and expect a consent, you know, within five seconds of me being handed it, then that’s not informed [laughter].” *P49, Parent, Declined, ON*

Time was a key factor as it created a space in which decisions were not rushed or made under duress.

“I think if they’re given that information in a low stress situation which is at the gynecologist’s office […], you know I don’t believe you should have to make your decision then and there to sign the paper […].” *P32, Parent, True Positive, NL*

Others indicated that time was a factor that was potentially prohibitive, and hence why a more informed consent approach was not adopted:

“Yeah I think because it would be a time issue and even if it just takes you, you know two minutes to explain what the test is for and what are the, you know, rates of false positives or whatever that’s someone’s time because you’re multiplying that by you know 2500 babies. So it’s not an insignificant amount of time and question then is “who is going to do that?” Like whose responsibility would it be to do that?” *P9*, *Healthcare Professional, NL*

Consequently, time, informational disclosure and professional responsibilities were associated, but also constrained within the system in which healthcare professionals had to operate.

### Information Ascertainment: Parent Responsibilities

While professionals were the main focus of discussion regarding roles and responsibilities, participants also indicated that consent approaches differed in the roles assigned to parents and the responsibilities that this created. In particular, when parents were expected to engage in a decision they were expected to review materials provided by professionals; it was their responsibility to ensure they were informed (when information was provided).

However, for some participants, parental responsibility extended under other models—a participant who distinguished presumed and implied consent indicated that under a presumed model it was a parental responsibility to seek information, whereas an implied consent model placed the onus on professionals.

“[…] presumed consent is something unless the parent has read up on it on their own, and…decides to go against it, it will happen.”*P22, Parent True Positive, ON*

### Information Ascertainment: Discussion and Understanding

For some participants, the provision of an information pamphlet did not mean parents had been informed. For these parents, disclosure included both the provision of information, but also the discussion and uptake of this information. As one set of parents suggested:

“[If] It’s just someone giving you a pamphlet, that’s not someone informing you that’s just someone telling you this is what’s here.[an] Informed decision was someone gave you the pamphlets, your gynecologist told you what was the benefits of it, then you made a decision whether to have it or not after that.” *P32, Parent, True Positive, NL*

For others, discussion was not specifically a requirement for an informed consent:

“Um. I think it’s [discussion with a healthcare professional to have questions answered] a bonus. I think that there’s enough information available to the parents” *P18, Healthcare professional, ON*

For some, a truly informed consent required the discussion to ensure that parents understood the information and any implications of their decision.

“Ah, informed means that … to me it means that you’ve communicated and have been understood. Right? And those are both loaded. Right? But what you need to communicate and be understood about … we’ve talked for hours about that before, but you know, I think to try and boil it down, I think, you know - what are the real risks if I do this; what are the real risks if I don’t; what are the benefits if I do this; what are the benefits if I don’t. Right?” *P56, Policy Decision-Maker, ON*

This most notably was discussed in the context of parent declines, and was on some occasions linked back to notions of voluntariness or rigor of the process. For some, there was an appropriate asymmetry of process between a simplified approach when parents agreed to screening, and a more rigorous checking of understanding when parents declined. This was justified on the basis of the potential implications of having a parent decline and then later having the child be identified as having a condition that could have been detected.

**Figure 1.**
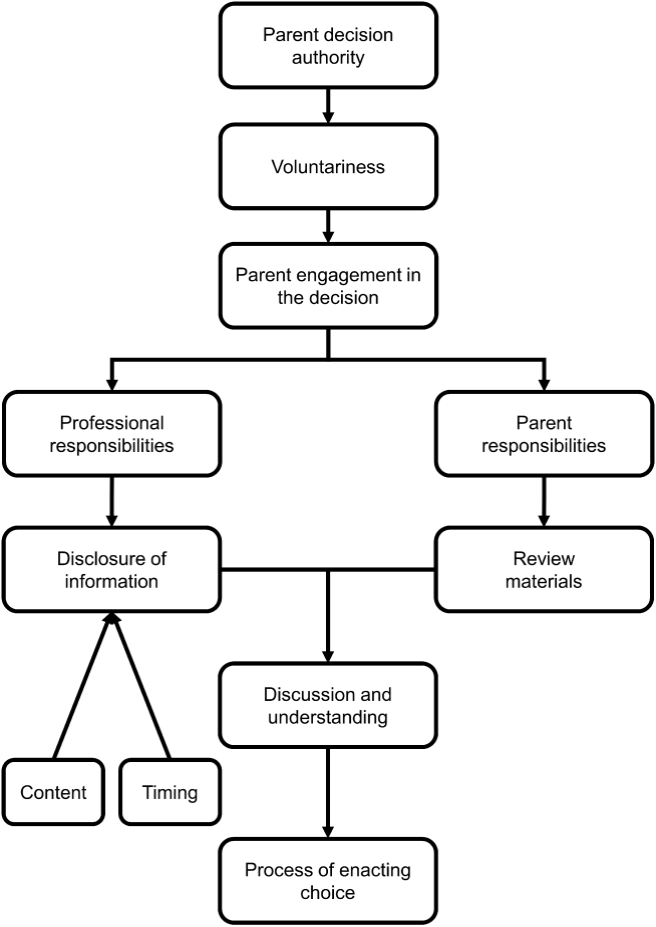
Schematic of the interrelationships of the identified factors.

While we have discussed these factors individually, Figure 1 illustrates the interrelationships. In general, perspectives on factors which have arrows leading to them will thus be informed by perspectives on the factor from which the arrow originates. However, given the varied conceptions noted, a particular perspective regarding one factor was not deterministic with respect to the perspective one would take on the subsequent factor. For example, our analyses indicate that perspectives on parental decision authority inform considerations regarding voluntariness and these perceptions in turn inform expectations of parent engagement with the decision to have their child screened. The individuals’ perspectives on the requirements of specific consent models regarding parent engagement thus informed expectations of disclosure, as well as the associated responsibilities of parents and professionals, expectations of parent understanding, and ultimately how consent was enacted. As such, we propose this as a model of wayfinding or definition-making, as opposed to specific combinations of factors that coalesce under particular consent-related terminology.

## Discussion

As NBS programs have evolved, so has discussion regarding the appropriateness of mandated screening. Numerous studies have been conducted exploring attitudes toward alternate approaches to consent (Campbell and Ross, 2003; Detmar *et al.*, 2007; Kerruish *et al.*, 2008; Dhondt, 2010; Hasegawa *et al.*, 2010; Miller *et al.*, 2010; Etchegary *et al.*, 2012; Moody and Choudhry, 2013), but few have explicitly considered the role of terminology. Our results indicate that consent-related terms are not consistently understood and that such inconsistencies have substantive implications for practice. We found variation in understanding of terms both within and between stakeholder groups, with no discernable systematic differences noted between the stakeholder groups.

Rather, we identified a number of key features in the stakeholder meanings of consent-related terms that were the source of varied meanings: (i) parent decision authority; (ii) voluntariness; (iii) parent engagement with decision-making; (iv) the process of enacting choice; (v) professional responsibilities (including disclosure of information and time to review); (vi) parent responsibilities; and (vii) the need for discussion and understanding prior to a decision. Indeed, the lack of systematic between-group differences supports our general proposal that these items may be common building blocks of definition-making.

However, our study must be considered within its limitations. While the present study provides empirical data regarding how the meanings of consent-related terms vary, we are unable to identify the degree to which this variation in meaning affects attitudes toward consent approaches or communication issues in practice. Future research should build upon our findings to quantitatively assess what the most prevalent perspectives are, and how individual meanings affect attitudes toward service delivery models and experiences of these.

### The Importance of Terminology

To date only a handful of studies have considered the role of language in relation to consent practices, despite it being well known that consent-related terminology is inconsistently used, particularly in newborn screening (Hargreaves *et al.*, 2005a,b). This ambiguity of terms should not be a surprise, given the noted semantic variation in the literature surrounding consent. Kleinig, for example, notes that:

“The semantic field of ‘consent’ is quite extensive, and includes terms such as ‘agreement’, ‘acquiescence’, ‘compliance’, ‘concurrence’, ‘willingness’, ‘connivance’, ‘condonation’, ‘accession’, ‘assent’, ‘submission’, ‘approval’, ‘permission’, ‘promise’, ‘authorization’, ‘consensus’, ‘concord’, ‘endorsement’, and so on. Within this semantic field, ‘consent’ often functions as a kind of carry-all, and, since the range of concepts implied is rather broad, different accounts appear to be indicated.” (Kleinig, 1982)

We suggest that the semantic differences are not benign. Rather, there are substantive consequences to differing perceptions of consent terms. This has previously been suggested in the way in which ‘mandatory’ has been used to describe screening programs in the USA, despite many allowing parents to opt out. As Wildeman and Downie (2001) note, the American Academy of Pediatrics Task Force stated that ‘for some parents, mandatory offering may be confused with mandatory screening’ (AAP Newborn Screening Task Force, 2000). This suggestion is consistent with previous work by Sutton, who found that Ontario policy decision makers disagreed on whether implied consent was the way screening occurred, whether implied consent is a voluntary or mandatory approach, and what an implied consent meant (Sutton, 2013). In our study, differences in the use of terminology had implications for information disclosure, documentation of consent as well as the roles that parents and professionals play in the consent process.

### Factors upon Which Conceptual Meanings Vary

The seven factors we identified as building-blocks of definition-making reflect elements of consent reported in the philosophy and screening literatures. While much of the work written on consent for newborn screening has tended to focus on disclosure-related aspects of consent or parental decision authority (Baily and Murray, 2008; Kerruish *et al.*, 2008; Hasegawa *et al.*, 2010; Miller *et al.*, 2010; Ross, 2010; Ulph *et al.*, 2017), our results indicate that parents’, professionals’ and policy decision makers’ conceptions are broader than this. Notably, our findings also illustrate an awareness of how ideals are constrained by existing social structures, such as time available, or how institutional liability concerns may require a written consent when a verbal consent may be deemed appropriate from a purely ethical perspective.

### Authority to Make Decisions—An Important Precursor to Voluntariness

In identifying factors upon which meanings differed our work develops the existing literature that has sought to discriminate axes upon which definitions of consent practices vary. Potter *et al.*, (2015), for example, argued that existing consent models can be differentiated along two lines: the authority given to parents in allowing or requiring them to make a decision and the expected level of parental engagement in this decision-making process. Hargreaves and colleagues differentiated consent approaches with respect to: degree of parental choice; information for parents; recognition of parental decisions; and parental choice and responsibility (Hargreaves *et al.*, 2005a).

Our results suggest that Hargreaves *et al*.’s element of ‘recognition of parental decision’ (Hargreaves *et al.*, 2005a) can be divided into two aspects: recognition in a moral sense that parents have decision-making authority over their child (as indicated through the availability of an option for their child not to be screened) and the physical act through which such a decision is conveyed; for example, verbally or in writing, what we term ‘Process of enacting choice’. We also suggest that while Hargreaves and colleagues present their definitional terms as existing upon a single continuum with associated changes within according to the relevant definition, our results indicate that stakeholders’ opinions do not conform to such a clean structure and that factors may be combined or recombined differently by different actors. As such, we consider our identified differences not as factors present or absent within a priori definitions, but as axes or waypoints in definition-making.

Our work also provides nuanced insights into existing constructs. One example was the suggestion that voluntariness is not binary, but exists on a continuum and is influenced by structural factors such as the ease to which certain actions could be invoked. Our results support prior observations that even when screening proceeds under a voluntary model, the presentation of information may not be consistent with an ambivalent position (Hargreaves *et al.*, 2005b; Nicholls, 2012).

### How Voluntary Must Voluntary Be?

A number of authors have argued that the nature of the testing-offer intrinsically brings some pressure to accept (Juth and Munthe, 2012), or discussed examples where decision-making options are constrained (Faden and Beauchamp, 1986; Corrigan, 2003; Halliday *et al.*, 2004). In the present study, participants raised an additional issue around which there is less consensus, namely, how voluntary does voluntary need to be? (Powers, 2007). Within the present study participants discussed how a decision to decline screening may lead to greater scrutiny of understanding than would occur if they had accepted, pointing to an asymmetry with respect to the perceived need to validate understanding based on the acceptance or decline of screening. Further, participants indicated that there may be greater or lesser degree of pressure to participate. This orientation—with greater focus on declining participation as opposed to agreement—is the inverse of traditional concerns for consent to research. In a research context, ignorance in declining participation is potentially viewed as less problematic—or at least is probed to a lesser degree—than ignorance in relation to participation (Bromwich, 2015). Rather, the asymmetry described by some participants in our study implied that when the default expectation is acceptance, then a choice to decline screening received a greater degree of attention, and potential evaluation with respect to whether such a decision was informed, than a potentially uninformed choice to accept screening. It remains a topic of discussion as to where legitimate attempts at informing and persuasion transgress, to use the language of Juth and Munthe (2012), into illegitimate infringement of autonomy. Bromwich, for example, has argued that the way in which information disclosure is handled may exhibit ‘illegitimate control’ over the voluntary nature of any consent (Bromwich, 2015; Bromwich and Millum, 2015; Bromwich and Rid, 2015). Hence, while screening may be voluntary, processes such as what information is provided or the administrative hurdles over which one must vault in order to decline, affect the voluntariness of the program to a greater or lesser degree. We see this as an important area for further study within NBS and, in particular, how comfortable stakeholders are with varying levels of evaluation of understanding.

### Disentangling Disclosure and Understanding

In the present study, the disclosure of information and the perceived need for understanding—together with the types and amount of information that needed to be understood—varied between participant meanings. The finding that these factors did not consistently co-occur within definitions of consent may lend support to conceptual work conducted in the context of consent for research participation by Sreenivasan (2003). This work, together with that of Bromwich (2015) and Bromwich and Millum (2015), disentangles information disclosure from the understanding of said information. Sreenivasan (2003), for example, has argued that, while comprehension of disclosed information is an ethical *aspiration*, it may not be an ethical *requirement*. Participants in the present study varied in their perceived needs for information to be understood, although—again—these views were not consistently aligned with one particular consent-related term or another.

Further, while these authors have readily attended to the obligations of professionals regarding disclosure, our present study builds on this literature by explicitly teasing out the role of parental responsibilities. In our proposed model (Figure 1), we suggest that any position taken regarding the responsibility of parents to read and comprehend the disclosed materials is contingent on the aforementioned perceptions regarding whether parents are expected to be engaged in the decision. Further conceptual work to unpack the role of parent responsibilities as a mediating factor between disclosure and the need for understanding would be beneficial. In particular, this would enhance the discussion from Sreenivasan (2003), Bromwich (2015) and Walker (2012), and whether there may be circumstances under which there would be greater expectation of understanding.

### Consequences of Variation: Substantive Not Merely Semantic

Our findings indicate that lexical variation surrounding consent-related terminology is not merely semantic, but reflects real substantive differences—such as the amount of information that needs to be disclosed, the degree of understanding required by parents and the responsibilities of healthcare professionals in their disclosure of information—requiring close consideration when developing screening program policies and practices. These substantive differences further point to the contextualized nature of the consent process; as Manson and O’Neill have argued ‘Information is not a context-independent “stuff” that flows from person to person’. (Manson and O’Neill, 2007). This context-dependent nature of consent was not only reflected in the discussion regarding the content of information disclosure, but also the varied reflections of the timing of information disclosure. This is consistent with previous research by Hayeems *et al.* (2009), which found that insufficient time was a commonly cited barrier to information provision.

Acknowledging that expectations vary depending on one’s conception of consent is important given the well-documented association between expectations, experience and satisfaction (Crow *et al.*, 2002). Where expectations, based on one’s conceptions of what a consent process may entail, are not met, parents may not only be dissatisfied but also could develop a negative relationship with the screening program. Our findings point to the need for transparency and clarity regarding what parents can expect under the consent process in place. Clear descriptions of what is discussed, and what parents can expect (or are expected to do) will serve to facilitate clear expectations. Having clear expectations, and disclosure processes that meet these expectations may improve satisfaction (Araia *et al.*, 2012). Further, as Potter *et al.* (2015) argue, meeting parent expectations regarding disclosure and discussion may not only engender trust but may also serve practical benefits of psychologically preparing parents should they receive a screen positive result. As such, we strongly suggest that NBS programs actively consider the seven factors identified when developing their consent policies. This, of course, requires ensuring that systems are in place to meet the educational needs of parents and families. This may require structural changes to current arrangements in order to support healthcare professionals in delivering information in a manner in keeping with parent’s needs. Table 2 presents our findings, together with implications for NBS practice.

**Table 2.** Domains and policy implications

Domain	Factor	Policy/practice implications
Decision-making	Parent decision authority	If parents have the authority to make decisions on behalf of their child (or not) this should be explicit. This may go hand in hand with the process of enacting choice such that it is made clear to parents both that they have the authority and how they can indicate their choice
	Voluntariness	While voluntariness may be assumed, procedures should be reviewed with respect to potential subtle pressures that may impinge on any voluntary decision-making. This may include the timing of decision-making or provision of materials that allow space for review. Further, if decisions are to be made voluntarily by parents this should be made explicit to parents
	Patient engagement with decision-making	If parents are expected to engage in decision-making (i.e. not be passive), then mechanisms need to be in place to both ensure materials reach parents, and that they are involved. This will require both resources and time, which may not be in place
	Process of enacting choice	Processes will need to be established or optimized for the capture of parent decisions. This may include new systems that again require resources and time
Information ascertainment	Professional responsibilities (including disclosure of information and time to review)	Materials and education that are in line with the consent process established may need to be developed
	Parent responsibilities	If parents have a responsibility to review materials, then there needs to be time afforded to them to review materials. If parents are not provided the time or opportunity to review materials then it denies this and they cannot be deemed responsible for not reviewing the materials. Thus, if consent processes are adopted under which parents are deemed to have a responsibility to review materials provided to them, then they must be given the time or opportunity to review materials
	The need for discussion and understanding prior to a decision	If there is an expectation that discussion and understanding is a prerequisite, then this will require time to have the discussion and resources that facilitate the discussion and understanding. Implicitly this is tied to the timing of information disclosure and establishing systems that ensure that information is disclosed in a timely fashion so as to allow review of materials and subsequent discussion to ascertain whether parents have understood

The seven identified factors and implications for NBS policy.

In the present study, informed consent processes were seen as fairly minimal for some participants while others perceived them as being detailed affairs, requiring extensive disclosure of information from healthcare professionals. This is problematic in a healthcare system that is unable to accommodate such time consuming approaches. If the suggestion from the present study holds (i.e. that the meaning of key terms varies between individuals), then the variation has the potential for substantial policy or practice implications if ambiguity leads to differing understandings of what is required vis-à-vis consent. This points to important practical questions regarding how best to develop service delivery models that attend to the potential variation in understanding, particularly within healthcare systems with limited resources.

We suggest that, despite the varied understanding of consent terminology, several recommendations can be made with respect to the consent process for NBS. To a great degree these relate to acknowledging the contextualized nature of the consent process and that expectations of engagement and disclosure will vary. In most jurisdictions parents will have decisional-authority over newborn screening for their child. Hence the immediate practical recommendations stem from acknowledging the varied understanding of terms, and implications for information disclosure and processes for enacting choice.

In our study, some participants viewed informed consent processes as fairly minimal while others perceived them as being detailed, requiring extensive disclosure of information from healthcare professionals. As a minimum, the healthcare system should acknowledge this and include space to provide more detailed discussion when needed. This requires time, but also core information that can be supplemented for those who wish to receive more. We acknowledge that this can be problematic in a healthcare system that is not set up to accommodate such time consuming approaches. However, if it is accepted that parents have the authority to make decisions for their child regarding NBS, then acknowledging and respecting this requires systemic approaches to support those decisions.

## Conclusion

Our data highlight important nuances to conceptions of consent-related terminology, indicate the practical implications of these noted differences, and reveal roles and responsibilities implied by approaches that have not been reported previously in the NBS literature. Future research to explore how variation in language can impact consent-related attitudes for newborn screening is needed and more work is required to explore how best to impart information to parents and develop systems that meet their needs.

## Supplementary Material

phz003_Supplementary_Data
