# Supplementary material for: What is in a Name? Parent, Professional and Policy-Maker Conceptions of Consent-Related Language in the Context of Newborn Screening
Source: Public Health Ethics. 2019 May 4;12(2):158–75. doi: 10.1093/phe/phz003 (PMC6655345; doi:10.1093/phe/phz003)
Supplement: phz003_Supplementary_Data [file phz003_supplementary_data.docx]

**APPENDIX 1: DRAFT Interview Guides for Mothers**

**Introduction/icebreaker**

1. How old is your infant? (If previous births how did it compare, differences etc?)

“Thank you very much for agreeing to help us with our study. As you may know, newborn screening – or the heel prick – takes place shortly after birth and involves a small amount of blood being taken from the baby. This blood is then tested for a number of conditions that, if undetected, can cause serious health problems.”

“What I’d like to do today is to talk to you about your experiences of this process, and touch on some of the issues that newborn screening raises and get your thoughts on these. We’re not trying to seek right or wrong answers, and anything you say will be kept confidential. Our hope is that we can draw on the experiences of mothers/fathers like yourself to make sure that the screening program operates as well as possible.”

**Experiences of NBS**

1. Had you heard of newborn screening/the heel prick/Guthrie test, before you had your most recent child?
2. Probe: If so who told you/how did you find out about it?
3. Probe: What do you recall being told?
4. Do you recall receiving information before [most recent child] being screened? [*Goes to informed aspect of consent*]
5. Probe: If yes, what sort of information was this (e.g. leaflet, discussion, etc.) who from, when, under what circumstances etc.?
6. Probe: If yes: and did you have chance to look at this information. If yes, what did you think of it? E.g., was it clear, did you understand it?
7. Probe: If no, what sort of information would you want? Did you ask for any information
8. How did you feel about the offer of screening? [*Goes to availability of choice/voluntariness of consent*]
9. Probe: Did you accept the test? If so, why. If not, why not.
10. How did you feel about deciding to have your child screened or not? [*Goes to ability to make a choice, or competency, at the time*].
11. Probe: What support did you have in deciding? Did you feel you needed support to decide?

**Attitudes toward consent for NBS**

“We’ve talked about your experiences of newborn screening with [most recent child]. I would like to now talk more generally about the consent process and your thoughts about this. People often talk about the need (or not) for informed consent for newborn screening.”

1. So, the first thing I want to ask is what “Informed consent” means to you.
2. Probe: E.g., What needs to happen for someone to give an informed consent?
3. Probe: Does this require particular types of information? Does this require people to understand certain things (rather than just be informed about them)? Do they need to make a decision or just a choice? [*Goes to exploring what people mean when they say “informed consent” especially in relation to parental decision authority and parental involvement in authorization*]

“In [ON/NL] the official mandate is for parents to be given information and then for screening to proceed unless the parent objects. There is no requirement for a parent to sign a form, although if they do decide not to have their child screened they may be asked to do so. This is sometimes described as ‘implied consent’”

1. How do you feel about this?
2. Probe: How does this compare to ‘informed consent’, what differences do you see and how do you feel about these differences?

“In several US states screening is mandated; that is parents are not given the option to decline.”

1. How do you feel about this?
2. Probe: How does this compare to ‘informed consent’ or ‘implied consent’, what differences do you see and how do you feel about these differences?

“Given what we have discussed about the different ways in which parents can take part in newborn screening:”

1. How do you think newborn screening *should* be provided? If you think there are parts of the different approaches described that are good please can you say what they are.
2. Probe: What is it about these aspects/components that is important?
3. Probe: If none of the approaches, how might you describe an appropriate approach?

**About you**

***This information will only be used to compare the answers from different people and will not be used by anybody else.***

**Age (*please check one only*):**

| Under 21 |  |
| --- | --- |
| 21-30 |  |
| 31-40 |  |
| 41-50 |  |
| Over 50 |  |

**19. Total number of children (*please check one only*):**

| 1 |  |
| --- | --- |
| 2 |  |
| 3 |  |
| 4 |  |
| 5 or more |  |

**20. Ethnic group (*please check one only*):**

| White |  |
| --- | --- |
| South Asian (East Indian, Pakistani, Sri Lanken, etc) |  |
| Black |  |
| Filipino/South Pacific |  |
| Latin American |  |
| Arab |  |
| South-east Asian (Vietnamese, Cambodian, Malaysian, Laotian, etc) |  |
| West Asian (Iranian, Afghan, etc) |  |
| First Nations/Métis/Inuit |  |
| Other, please specify___________________________________ |  |

**21. What language do you speak at home? (please check all that apply)**

| English |  |
| --- | --- |
| French |  |
| Other, please specify_____________________ |  |

**22. Highest level of educational attainment (*please check one only*):**

| None |  |
| --- | --- |
| Elementary school |  |
| High school diploma |  |
| Community college, technical college or CEGEP |  |
| University degree – undergraduate |  |
| Professional degree (e.g. education) |  |
| University, graduate degree or higher |  |
| Other, please specify_____________________________________ |  |

**Please tick the box if you would like a summary of the findings when complete**

**APPENDIX 2: DRAFT Interview Guides for Healthcare Professionals**

“Thank you very much for agreeing to help us with our study. As you may know, newborn screening – or the heel prick – is almost universal with respect to coverage and uptake, not only in Canada but throughout the world. As you may know, the expansion of newborn screening to include conditions that do not meet traditional criteria for inclusion in screening programs, has led to a great deal of discussion about how screening should proceed.”

“What has been lacking is comparative work with key stakeholders and a failure to explore how interpretations terms such as ‘informed consent’ affect attitudes toward consent practices and in turn how these attitudes affect experiences.”

“What I’d like to do today is to talk to you about your experiences of the newborn screening process, and touch on some of the issues that newborn screening raises and get your thoughts on these. Our hope is that we can draw on the experiences of healthcare professional like yourself to inform delivery and policy development.”

**Introduction/icebreaker**

1. Can you tell me a little about how newborn screening works in [ON/NL] and how the offer of screening generally proceeds?

**Experiences of NBS**

1. Can you describe you common involvement in the consent process for newborn screening?
2. Probe: How often are you involved in this aspect of newborn screening/parental-child care? E.g. daily, weekly, only rarely?
3. Probe: Do you see this as a major or minor component of your role in the parent-child care pathway?
4. Probe: What specific aspects are you involved with? E.g. information delivery, discussion, declines?
5. What are the practicalities of offering screening in [ON/NL]? [*Goes to availability of choice/voluntariness of consent*]
6. Probe: What are the barriers to implementing the screening offer? E.g. Some studies indicate time pressures that preclude appropriate consent approaches
7. Probe: What aspects of delivery are helpful? Have there been changes that have helped?
8. How do you feel about approaching parents about newborn screening? [*Goes to ability to make a choice, or competency, at the time*].
9. Probe: Was training provided? Did you feel you needed training?
10. Have you ever experienced a parent decline newborn screening?
11. Probe: How did that make you feel? Did you feel prepared to cope with this situation?
12. Probe: How did you respond? Did you feel comfortable responding? Do you feel this is a role for other professionals (e.g. lawyers)?
13. Probe: How was the situation resolved?

**Attitudes toward consent for NBS**

“We’ve talked about your experiences of newborn screening. I’d like to now talk more generally about the consent process and your thoughts about this. People often talk about the need (or not) for informed consent for newborn screening.”

1. So, the first thing I want to ask is what “Informed consent” means to you.
2. Probe: e.g. What needs to happen for someone to give an informed consent?
3. Probe: Does this require particular types of information? Does this require people to understand certain things (rather than just be informed about them)? Do they need to make a decision or just a choice? [*Goes to exploring what people mean when they say “informed consent” especially in relation to parental decision authority and parental involvement in authorization*]

“In [ON/NL] the official mandate is for parents to be given information and then for screening to proceed unless the parent objects. There is no requirement for a parent to sign a form, although if they do decide not to have their child screened they may be asked to do so. This is sometimes described as ‘implied consent’”

1. How do you feel about this?
2. Probe: How does this compare to ‘informed consent’, what differences do you see and how do you feel about these differences?

“In several US states screening is mandated; that is parents are not given the option to decline.”

1. How do you feel about this?
2. Probe: How does this compare to ‘informed consent’ or ‘implied consent’, what differences do you see and how do you feel about these differences?

“Given what we have discussed about the different ways in which parents can take part in newborn screening:”

1. How do you think newborn screening *should* be provided? If you think there are parts of the different approaches described that are good please can you say what they are.
2. Probe: What is it about these aspects/components that is important?
3. Probe: If none of the approaches, how might you describe an appropriate approach?

**APPENDIX 3: DRAFT Interview Guide for Policy Decision-Makers**

“Thank you very much for agreeing to help us with our study. As you may know, newborn screening – or the heel prick – is almost universal with respect to coverage and uptake, not only in Canada but throughout the world. As you may know, the expansion of newborn screening to include conditions that do not meet traditional criteria for inclusion in screening programs, has led to a great deal of discussion about how screening should proceed.”

“What has been lacking is comparative work with key stakeholders and a failure to explore how interpretations terms such as ‘informed consent’ affect attitudes toward consent practices and in turn how these attitudes affect experiences.”

“What I’d like to do today is to talk to you about your experiences of the newborn screening policy development process, and touch on some of the issues that newborn screening raises and get your thoughts on these.”

**Introduction/icebreaker**

1. Can you tell me a little about how newborn screening policy works in [ON/NL] and your role within this decision-making process?

**Experiences of NBS**

1. Can you describe your involvement in the decision-making process for newborn screening in [ON/NL]?
2. Probe: How does [committee/decision-making structure] convene? How often are you involved in this aspect of newborn screening E.g. Monthly, quarterly, only rarely?
3. Probe: Do you see this as a major or minor component of the elements discussed in relation to newborn screening? E.g. is it more technical issues?
4. Probe: Have you taken on any particular roles in this broader decision-making capacity?
5. Can you describe the current policy in [ON/NL] in relation to parent participation in newborn screening?
6. Probe: How was that determined?

**Attitudes toward consent for NBS**

“We’ve talked about your experiences of developing policy for newborn screening. I’d like to now talk more generally about the consent process and your thoughts about this. People often talk about the need (or not) for informed consent for newborn screening.”

1. So, the first thing I want to ask is what “Informed consent” means to you.
2. Probe: e.g. What needs to happen for someone to give an informed consent?
3. Probe: Does this require particular types of information? Does this require people to understand certain things (rather than just be informed about them)? Do they need to make a decision or just a choice? [*Goes to exploring what people mean when they say “informed consent” especially in relation to parental decision authority and parental involvement in authorization*]

“As you describe, in [ON/NL] the official mandate is for parents to be given information and then for screening to proceed unless the parent objects. There is no requirement for a parent to sign a form, although if they do decide not to have their child screened they may be asked to do so. This is sometimes described as ‘implied consent’”

1. How do you feel about this?
2. Probe: How does this compare to ‘informed consent’, what differences do you see and how do you feel about these differences?

“In several US states screening is mandated; that is parents are not given the option to decline.”

1. How do you feel about this?
2. Probe: How does this compare to ‘informed consent’ or ‘implied consent’, what differences do you see and how do you feel about these differences?

“Given what we have discussed about the different ways in which parents can take part in newborn screening:”

1. How do you think newborn screening *should* be provided? If you think there are parts of the different approaches described that are good please can you say what they are.
2. Probe: What is it about these aspects/components that is important?
3. Probe: If none of the approaches, how might you describe an appropriate approach?
